# On Dropsies Connected with Suppressed Perspiration and Coagulable Urine

**Published:** 1836-07

**Authors:** 


Art. XVI.—*On Dropsies connected with suppressed Perspiration, and coagulable Urine*, by Jonathan Osborne, m.d., President of the King and Queen's College of Physicians, in Ireland, Physician to Sir P. Dun's and Mercer's Hospitals, &c. Pp. 64. 1835.

A regret expressed by Dr. Gregory several years ago, that the connexion between albuminous urine and organic disease of the kidney, pointed out by Dr. Bright, had not attracted that degree of attention which its frequency and importance would seem to demand, is now repeated by Dr. Osborne, who condemns, at the same time, in severe terms, the conduct of some who have depreciated the discovery by loose statements, instead of resisting it with facts. The importance of the subject itself, and the suspicion that it has been neglected or unfairly dealt with, are our reasons for taking a survey of the evidence which has been adduced on both sides.

Dr. Bright described in 1827, in his Medical Reports, an organic change in the kidney of many dropsical patients, commonly consisting (when fully developed) of a deposition of a yellowish granular matter in its texture, which he invariably found connected with an albuminous state of the urine during life. He hazarded the conjecture that there were three forms of this organic disease. In the first the kidney was soft, of a mottled yellow externally; the cortical part yellowish grey, and the tubular pale; in the second stage the cortical part was converted more or less into a granulated texture with a copious white interstitial deposit; in the third these granulations were increased, of a yellow, red, or purplish colour, projecting externally so as to render the surface of the kidney rough, and producing a semi-cartilaginous hardness of the whole gland, the tubular portions being drawn near the surface. This disease was often indicated by pain across the loins, and upper part of the abdomen, and by sickness and vomiting. During its progress there was a strong tendency to inflammations, especially of the serous, and sometimes of the mucous membranes, and to effusions of blood and serum into the brain. The specific gravity of the urine was very low, owing to a deficiency of urea and salts. He considered it most probable that this organic change was secondary: being the result of various hurtful causes influencing it through the medium of the stomach and skin. The great tendency to inflammation of other organs led him to recommend bloodletting, and cupping the loins to relieve the local disease. He preferred supertartrate of potash, but employed occasionally all diuretics except mercury.

The accuracy of Dr. Bright's views was put to the test by Drs. Christison and Gregory, in the Edinburgh Infirmary; and they subsequently published eighty-seven cases, strongly confirming the connexion between the organic disease and the condition of the urine: in every instance in which an examination took place after death, the granular state of the kidney being found. Dr. Gregory, however, showed that dropsical effusion was not an essential, though a prominent symptom; for, out of eighty cases, there were twenty-two in which it was absent. Dr. Osborne, in the treatise now before us, powerfully supports Dr. Bright's opinion, to which he has become a convert "only by virtue of a long series of observations, many of which were instituted in the expectation of overthrowing it." As physician to Sir Patrick Dun's Hospital, his opportunities have been considerable. In the first part of the present treatise, published in the Dublin Journal, in January, 1834, he states that eighty-four cases had fallen under his notice of coagulable urine.

"Of this number examinations after death evince the disease of the kidneys in nine cases; while the remaining cases prove the existence of the same disease, so far as it is susceptible of proof, by similarity of symptoms, of cause, of collateral circumstances, and of *adjuvantia* and *lædentia*; and I can with truth aver, that I have witnessed many more cases which are not sufficiently detailed for the present occasion, but which, without any exception, corroborated the truth of Dr. Bright's proposition. The negative evidence in my possession is too copious to be detailed. It is, however, decisive as to the question at issue. In consists of numerous cases of dropsies, connected with diseased liver, impediments of circulation, or respiration, or general debility, which terminated fatally, in which the urine was examined before death, and found not to coagulate, and the kidneys were found to be free from disease; also cases ending fatally, but unconnected with dropsy, in which the kidneys were healthy, and the urine did not coagulate. This evidence appears to me peculiarly valuable, inasmuch as during the last three years I have anxiously sought for every opportunity of examining the kidneys of every individual in whom the urine had been examined during life; and in no one instance have I met with coagulable urine without diseased kidneys, or healthy kidneys with coagulable urine." (P. 13.)

In January, 1835, the second part of Dr. Osborne's Essay was written, and he says:

"Since that time I have continued to enjoy the same opportunities of observation as before; and, though I have anxiously sought for evidence, either adverse or favorable to this opinion, I have not met with a single instance of urine coagulating in a constant manner, in which an opportunity of examination after death was afforded, that did not present the disease of the kidney; nor, on the other hand, an instance of the disease being found in the kidney after death, in which, on taking a specimen of the urine in the bladder, it did not coagulate." (P. 21.)

It is curious to observe how Dr. Bright's views have been confirmed by authors who preceded him, who incidentally mention the two circumstances, but who failed to trace the connexion. Thus, Dr. Wells and Dr. Blackall detail the history and dissections of nine cases of dropsy in which albuminous urine existed during life, and the kidneys were found diseased; but preconceived notions of the nature of dropsy prevented these physicians from tracing the connexion.

Such is the evidence in support of Dr. Bright's opinion, and the only facts which have been adduced against it are three cases published by Dr. Darwall. In two of these there was albuminous urine, and examination after death showed that "the kidneys were perfectly sound" in one, and in the other "the cortical part was paler," but without change of structure. In the third there was uncoagulating urine, although the kidneys were in the first stage (according to Dr. Bright) of organic disease: this patient however (as Dr. Osborne justly remarks) was scrofulous: now, as paleness of the internal organs frequently is observed in such cases, and as the first stage is with difficulty distinguished from the natural state, this instance does not carry any weight as an objection. In the second case the cortical part was paler than natural, and therefore not perfectly healthy. The first case consequently is the only one which seems to be an exception. The specific gravity of the urine is not mentioned.

Objections have been made on other grounds; such as the existence of albumen in the urine of those in perfect health, or after taking indigestible food, or mercury: but, if albumen is an ingredient in healthy urine, it does not exist in sufficient quantity to be coagulable by heat, which was invariably the case in this disease; and the other objections are founded on the *transitory* appearance of albumen, not on its *continuous* secretion in connexion with urine of low specific gravity. The coexistence of anasarca with coagulable urine after scarlatina has also been adduced to prove that the albuminous secretion cannot be fairly regarded as the consequence of organic disease, as this complication is so often a transitory complaint of little danger. The state of the kidney under such circumstances has not been sufficiently attended to, but, as far as the evidence goes, it is in favour of Dr. Bright's views. In a paper on scarlatina by Mr. Hamilton, in the Edinburgh Medical Journal,* several fatal cases of dropsy following this eruption are related, in all of which the

* Vol. 39. P. 154.

kidneys were found in that yellowish mottled condition which marks the first stage of the organic change: more than one teacher of reputation is reported to have denied the connexion which Dr. Bright endeavoured to establish, when it was first promulgated; but the general terms in which these objections were couched, and the difficulties which invariably beset the admission of novelties when contradictory of firmly fixed opinions, render such statements of no weight in any estimate of the evidence bearing on this question. As our readers are now in possession of the facts on both sides, they can make their own decision. In forming, however, a decision, the kind of proof which a subject admits of should always be borne in mind. If it is considered necessary to establish the universality of the fact in order to its belief, a solitary exception, if well attested, and the limited number of cases, would be serious arguments for withholding our assent; but, on the other hand, if we are contented with as much evidence as can be procured in like cases and as the subject admits of, we shall give that qualified assent to the proposition which is sufficient for all practical purposes. We are not aware that the connexion between any one organic change and a sign or symptom has been established as an universal fact,—that there is one (so called) pathognomonic symptom in the rigorous acceptation of the term. Exceptions to the best established become more numerous the farther we advance in pathological investigations. Pneumonia, for instance, may occur without its characteristic expectoration, and even without expectoration at all; pleurisy without its fixed and pointed pain; and one half of the brain may become converted into a vast abscess without any appreciable disturbance in the intelligence. Under such circumstances we get over the difficulty by attributing it to idiosyncrasy, and, although the meaning attached to this term is not always very precise, and its application not sufficiently restricted, yet it serves the useful purpose of preventing the exception from rendering nugatory the vast body of affirmative evidence.

Regarding this kind of evidence as sufficient, because no other is attainable, it scarcely requires to be said that we think Dr. Darwall allowed an undue weight to the very few apparent exceptions which fell under his immediate cognizance, considering the mass of evidence on the other side which Guy's Hospital and the Edinburgh Infirmary had furnished; but that he should have done so is not surprising, as the exceptions happened at the very commencement of an enquiry into the truth of an opinion altogether novel to one who had studied the subject deeply and practically, but who had overlooked the connexion, and therefore was disinclined to believe it. Dr. Osborne candidly tells us that he himself began the examination of this question with a view to overthrow it, certainly a state of mind the most opposite to that with which a searcher after truth should begin his investigations; and, although we agree with him in his general conclusions, yet it strikes us that the manner in which he has argued the subject is marked with the same temper to which he confessed when he began his enquiries, as it savours more of the partisan than of the calm enquirer. On this account among others, we have preferred making our own statement of the points at issue, to reproducing Dr. Osborne's arguments.

The second part of Dr. Osborne's work is devoted to the consideration of the causes of this organic disease of the kidney, and its treatment. We have previously mentioned that Dr. Bright considered it probable that the renal disease was secondary, resulting from various hurtful causes influencing the kidneys through the medium of the stomach or skin. Dr. Osborne considers, from the following reasons, that it is produced most frequently by a suppression of the healthy secretion of the skin.

"The force of the circulation in this disease appears to be depressed by the action of some specific agency not as yet ascertained. The surface and extremities are uniformly cold—the latter being either livid or pallid; and, on reviewing my collection of cases, I find that in all of them the pulse was low, undulating, and ranging from sixty to ninety, except when they were complicated with inflammations; and that in those cases it was considerably less frequent than usual.

"The perspiration was either completely extinct, or confined to occasional breakings forth in the head or chest, the palms of the hand, or soles of the feet. The skin was dry and shining, harsh to the touch; and, on examining it with a lens, the usual eminences belonging to the orifices of the follicles were no longer to be found, and the orifices themselves were hardly perceptible, except when they appeared like black dots, in consequence of being filled with the residue of old secretions.

"Whenever general perspiration came on, either spontaneously or in consequence of medicine, then the cases always terminated favorably." (P. 26.)

"On reviewing the causes of the disease in thirty-six cases, in twenty-two individuals it could be directly referred to suppressed perspiration. One of these was Thomas Leahy, a remarkably vigorous man, in his thirty-fifth year, of sober habits. It appears that he was inconvenienced by the excessive perspiration of his feet, and that at the suggestion of a friend he wore fuller's earth in his shoes in order to repress it. The effect was immediate. The perspiration ceased not only in his feet, but also in every part of his body. Diarrhœa soon came on; and, when this was subdued by appropriate remedies, universal œdema, with coagulable urine, succeeded. Although, under the treatment adopted, the œdema was removed, yet the healthy action of the skin was never restored, and I am informed that his dropsy returned. In another of those cases the commencement of the disease was attributed to cold bathing: but the most frequent cause of it was remaining in wet clothes." (P. 32.)

The large quantity of perspiration constantly excreted in the healthy condition, and the immediate effect of cold in obstructing this, and in producing an increased secretion of urine, are adduced as additional reasons for supposing that any permanent suppression of perspiration would produce permanent irritation of the kidneys.

"The next frequent cause is the abuse of diuretic drinks and medicines. Of the thirty-six cases, ten occurred in confessed drinkers of ardent spirits." * *

"Diuretic medicines also have appeared to me to be a frequent cause of the disease. Squills and the diuretic salts, although of the utmost importance in many affections of the thorax, yet when long continued, as they often are after the true indications for their use have ceased, become the means of bringing such on again by producing over-excitement of the kidney, and this disease as a consequence." (P. 33.)

The close scrutiny which is necessary to detect the first deviations from health, and the few opportunities which are afforded to medical men of personal observation of these initiatory states of disease, account for the little accurate knowledge we possess on this most important branch of medical investigation; but the same reasons render caution advisable in believing new opinions on these matters. The views of Dr. Osborne on the causes of this organic renal disease have the great merit of leading to and explaining the rationale of a judicious and successful plan of treatment. The coincidence of dry skin with the diseased secretion of urine, and the restoration of the skin to its normal state by warmth and baths bringing back the healthy secretion of urine, as well as the production of the disease very frequently by causes tending directly to suppress the perspiration, are strong, though not altogether conclusive, arguments. The intimate sympathy between the skin and the kidneys may explain the coexistence of unhealthy conditions of their functions, and the effect which remedies influencing the one function may exert on the other; whilst there may be a *tertium quid*, on which both the affection of the skin and of the kidneys mutually depend. In diabetes, for instance, there is a dry skin with a diseased secretion of urine, and a meat diet will diminish the quantity of urine, and improve its quality, whilst at the same time the skin becomes relaxed and moist; both the secretion of urine and of perspiration apparently depending (as far at least as can be judged from the "juvantia,") on some disorder of the assimilative functions. To determine the question satisfactorily, proofs are necessary that an impaired state of the function of the skin precedes for some time the albuminous condition of the urine; for the bare fact, that causes which (among other tendencies,) check the perspiration frequently produce the disease, is insufficient evidence of its initiatory state. In estimating also the value of the proof resting on the effect of the diaphoretic remedies, it must be remembered that treatment directed to so extensive a surface of the skin must have a powerful effect on any local disease, whatever may have been its cause.

To the same cause, obstructed perspiration, Dr. Osborne attributes the inflammatory state of the mucous membranes which he found to coexist so frequently with this disease. Thus,

"Of the thirty-six patients, eighteen laboured under bronchitis in different degrees of intensity; eleven had gastro-enteritic inflammation, denoted by thirst, vomiting, or diarrhœa; and the two diseases were in six instances combined in the same individuals. Thus it appears that nearly two-thirds of the entire number laboured under inflammation of the mucous membranes." (P. 35.)

The coexistence of affections of the mucous membranes has been remarked by other observers. Dr. Gregory particularly mentions diarrhœa and vomiting as among the most constant symptoms; in forty-six patients out of eighty, these were generally troublesome, frequently urgent, and untractable symptoms; but he differs from Dr. Osborne in his opinion as to their cause, as he distinctly states that they existed "without any distinct signs of inflammatory action."*

The comparison of this disease with diabetes leads, we think, to a more satisfactory explanation of the nature of the concomitant affections of the mucous membranes than has hitherto been given. There are many points of analogy between the two diseases: thus, both are frequently produced by cold so applied as to check perspiration; in both there is a dry skin, with a disordered secretion of urine; and in both the restoration of the perspiration is a great step towards the cure. In diabetes, however, the quantity of urine is greatly increased; for as the skin no longer perspires, and the bowels are either constipated or not relaxed, the only exit for

* Edinburgh Medical and Surgical Journal. Vol. 37, p. 79.

superfluous secretion is through the kidneys: but, in the disease under consideration, neither the skin nor kidneys are outlets for the superfluous matter; for the skin no longer acts, and the urine, which is often scanty, is always deficient in solid matters (salts and urea); by means of the dropsical effusion some exit is obtained, but it is far from improbable, we conceive, that by the increased secretions from the mucous surfaces, producing diarrhœa and catarrh, the superfluous matter, which, under natural circumstances, would have passed off by the skin or urine, is evacuated from the system. The examination of forty-four fatal cases of this renal disease, reported by Dr. Gregory, in order to elucidate this matter, is most decidedly in favour of the view we have here taken of the nature of these excretions. The cases are divided into three sets: the first division contains fifteen cases of dropsy in which diarrhœa and vomiting were prominent symptoms; the second includes nine cases in which the thoracic organs were affected; and of these there was bronchial expectoration in seven, generally profuse; one died from phthisis, and the other from acute pneumonia. The third division comprehends twenty cases of albuminous urine and diseased kidney without dropsy; in sixteen of these, diarrhœa, vomiting, and bronchial affections were prominent symptoms; and the four remaining cases were inconclusive either way, as two died from typhous fever, one after lithotomy, and the other is superficially reported. Further proofs could be brought from physiology, such as the general analogies between the skin and mucous membranes; and, as far as the mucous membrane of the lungs is concerned, the fact which has been recently so conclusively proved by the experiments of Professor Tiedemann,* that the lungs not only perform certain chemical changes on the blood, but act as a genuine secretory organ for the venous blood, by throwing off the volatile and unassimilable particles. If, therefore, these secretions from the mucous surfaces, so constantly coexisting with this disease, are the channels through which those superfluous excretions are evacuated which naturally are got rid of by the skin and kidneys, it follows that the restoration of the functions of the skin will be one of the best means of relieving the diarrhœa or catarrh; and the obstinacy of these symptoms to common remedies directed to their removal during the existence of the primary disease is explained.

The inefficacy of common diuretics seems to have first induced Dr. Osborne to change his remedies.

"When a patient was placed under my care, with general œdema, coagulable urine, and dry skin, I directed him to be kept in bed, in order to maintain warmth of the surface, which is usually disposed to be cold. It has happened frequently that, by external heat alone, an improvement both in the quantity and quality of the urine, and a material subsidence of the œdema, have taken place. The first medicine ordered was usually a purgative; and in the choice of this, in order to avoid ambiguity as to its mode of action, I abstained from the use of all those articles which are reputed diuretic; such as compound of jalap, or supertartrate of potash; and I generally employed the senna mixture. I then commenced a diaphoretic course, by administering foot baths, hip baths, or general baths; the last either of water or of vapour, according as they appeared to agree best with the individual case, at night at

* No. I. of this Journal; p. 241.

the hour of going to bed. The patient also took at night eight grains of pulv. jacob. ver. 4. of pulv. ipecac. c. opio, and ten grains of confect. aromat.

"The usual drink was barley-water. In case, however, of tendency to stupor, or headach, the Dover's powder was omitted, or given in smaller doses. In one case, in which no perspiration was produced by the above and other means, it followed the use of the following mixture: ℞. Aq. Acet. Ammon. ℥iv. Sulphur. Subl. ℥j. Vini Ipec. ʒj. Ext. Opii aq. gr. ij. Aquæ Fœnic. dulc. Syrup. Sacch. empyreumat. utriusque ℥ij., one ounce to be taken every hour.

"When the vapour bath was not attended by perspiration, from want of reaction on the part of the patient, he was directed to take, while in it, two drachms of the Tinct. Guaiaci Ammoniat; when, however, (as sometimes happened,) both vapour and water baths produced coldness of the extremities, they were discontinued." (P. 42.)

"When there was a continued tendency to coldness of the surface, unaccompanied by feeble action of the heart, the diaphoretic preferred was Tinct. Guaiaci Ammoniat. ʒij. Sulphuris Loti ʒj. Mist. Camph. ℥j. Sp. Piment. ʒss., or the following: ℞. Carbon. Ammon. ʒss. Mist. Camph. ℥vj., an ounce to be taken every two hours. In connexion with these remedies, administered in the evening with a view to procure a perspiration during the hours of sleep, warm applications were kept up during the day, and frequently a succession of bags of hot salt was maintained, when the heat of the extremities could not be otherwise preserved. When perspiration was restored in one part of the body, as in the trunk, but not in the limbs, the latter were rubbed several times during the day with an infusion of two drachms of bruised mustard seeds in distilled vinegar, with Naphtha, or some other suitable stimulating embrocation.

"Having never failed in removing this kind of general dropsy whenever the entire surface of the body was restored to a perspiring state, it is not surprising that I should bestow the utmost attention on this part of the treatment." (P. 44.)

"Next in importance to the restoration of the function of the skin, and indeed in most cases expedient, as contributing to that great object, was bloodletting." (P. 46.)

Where the indications were less decided, cupping the loins or leeching was employed; also cautious counter-irritation by means of lint steeped in tinc. lyttæ, and covered with oiled silk, a cleanly and rapid mode of vesicating. These were applied in rapid succession or kept open with iodine treatment. The common purgatives were given when necessary, and administered in the morning so as not to interfere with the diaphoretics. As relapses from exposure to cold may take place, flannel should be worn next to the skin, and baths and frictions used in case of dryness of the skin recurring; residence also in a warm climate is very advisable. Exercise should be carried to perspiration, if the strength permits, and bandages applied to the legs during convalescence. Complications require additional means. In dry bronchitis, copaiba and tincture of cubebs in small doses were advantageous; when the expectoration was copious for a long time, and impeded respiration, one grain of acetate of lead and a quarter of a grain of opium diminished the secretion and irritation. Leeches to the larynx unloaded the bronchial tubes, causing a cessation of cough and dyspnœa. Irritation of the stomach and bowels was relieved by leeches, warm applications, and a diet of rice and arrowroot. Dysentery commencing by tenesmus and general excitement was most speedily removed by an enema of four grains of nitrate of silver and eight ounces of water, followed in three hours by a starch enema with laudanum. In pericarditis, tartar emetic internally, with topical and general bloodletting, amended all the symptoms; and in valvular disease of the heart, a mixture of a small quantity of tincture of digitalis, with carbonate of ammonia, camphor, and Hoffman's liquor, was followed by diminution of the violent action of the heart, a sense of general relief, and a capability of sleeping with comfort at night. In diseased aortic valves, a large issue over the region of the heart was of the highest importance. When ascites follows chronic peritonitis or indurated liver, and intractably remains after the general œdema is removed, Dr. Osborne recommends the following measures, which in his hands have rendered tapping seldom necessary. They may be continued when mercury and drastic purgatives are abortive, and the declining strength of the patient forbids such powerful remedies.

"These are the repeated application of leeches to the rectum, so as to unload the vessels of the vena portæ. The applications of various stimulants to the abdomen, as, 1st, an ointment composed of equal parts of iodine, mercurial, and cantharides ointments. 2dly. A paste formed of Spanish soap, spread upon linen, and sprinkled over with muriate of ammonia immediately before being applied; which, by the chemical decomposition that ensues, and the consequent gradual extrication of ammonia, produces heat and redness; 3dly. Sinapisms, suffered to remain till the pain becomes urgent. These have the advantage of healing with great rapidity. 4thly. Frictions of six or more drops of croton oil. These are, however, rather uncertain; in some individuals producing no effect, and in others followed by erysipelas, extending beyond the seat of the application. 5thly. A mixture composed of one part of tincture of digitalis, and two of aquæ muriat calcis; a teaspoonful to be rubbed on the abdomen, morning and evening. This compound appears to excite the absorbents, and increases the discharge from the kidneys, but does not produce any sensible redness of the skin." (P. 57.)

Tinnitus aurium, watchfulness, delirium, stupor, or headach, with increased heat of the head, are formidable symptoms, as death is frequently produced by a low form of arachnitis. Under such circumstances, Dr. Osborne recommends, besides bleeding from the temporal artery and by leeches, the free exhibition of calomel followed by brisk purgatives.

The length to which this notice has extended itself may appear to be disproportionate to the unpretending size of the work, but it furnishes more matter for an analytical notice than its appearance would indicate, for the results of the cases are only given, "as the details of cases (says Dr. Osborne,) attract little attention, and are in fact seldom read;" which, though perfectly true, is we think, notwithstanding, an insufficient reason for the omission; for, although the generality of readers may, either from want of time or from indolence, find it convenient to embrace conclusions without the trouble of reflection or examination, yet the careful student consults the details to settle doubtful points, and not unfrequently finds them of the highest importance as confirming new views which had escaped the observation of the original reporter. Thus, for instance, Dr. Osborne confirmed his own observations on the inefficacy of diuretics in this form of dropsy by a careful examination of the cases detailed by Dr. Gregory for quite a different object.

In conclusion, we have no hesitation in expressing our conviction that Dr. Osborne's work evinces much good sense, correct judgment, and considerable powers of observation; and that the plan of treatment he proposes is rational, judicious, and eminently calculated to prevent indiscriminate attempts to make the kidneys in all cases the channels by which dropsical effusions are to be evacuated.

